# Coming of age, becoming obese: a cross-sectional analysis of obesity among adolescents and young adults in Malaysia

**DOI:** 10.1186/s12889-016-3746-x

**Published:** 2016-10-13

**Authors:** Christopher Pell, Pascale Allotey, Natalie Evans, Anita Hardon, Johanna D. Imelda, Ireneous Soyiri, Daniel D. Reidpath, The SEACO Team

**Affiliations:** 1 Centre for Social Science and Global Health, University of Amsterdam, Nieuwe Achtergracht 166, 1018 WV Amsterdam, The Netherlands; 2 South East Asia Community Observatory (SEACO), 6th Floor, Wisma Centrepoint, Jalan Sia Her Yam, 85000 Segamat, Johor Malaysia; 3 Global Public Health, Jeffrey Cheah School of Medicine and Health Sciences, Monash University Malaysia, Jalan Lagoon Selatan, 47500 Bandar Sunway, Selangor Darul Ehsan, Malaysia; 4 EMGO Institute for Health and Care Research, VU University Medical Center, Van der Boechorststraat 7, Van der Boechorststraat 7, 1081 BT Amsterdam, The Netherlands; 5 Department of Social Welfare, Faculty of Social and Political Sciences, University of Indonesia Puri Depok Mas Blok P-39, Depok, 16436 West Java Indonesia; 6 Centre for Population Health Sciences, Medical School, University of Edinburgh, Teviot Place, Edinburgh, EH8 9AG Scotland

**Keywords:** Obesity, Malaysia, Overweight, Adolescents, Physical activity

## Abstract

**Background:**

Malaysians have become increasingly obese over recent years. The transition from adolescence to early adulthood is recognized as critical for the development of eating and activity habits. However, little obesity-related research focuses on this life stage. Drawing on data from a health and demographic surveillance site in Malaysia, this article describes obesity and overweight amongst adolescents and young adults in a multi-ethnic population.

**Methods:**

Data were collected at the South East Asia Community Observatory (SEACO) in Segamat District, Johor. In this dynamic cohort of approximately 40,000 people, 5,475 were aged 16–35 in 2013–2014. The population consists of Malay, Chinese, Indian and Indigenous (Orang Asli) families in proportions that reflect the national ethnic diversity. Data were collected through health profiles (Body Mass Index [BMI] measurements in homes) and self-report questionnaires.

**Results:**

Age and ethnicity were associated with overweight (BMI 25.0–29.9Kg/m^2^) and obesity (BMI ≥ 30Kg/m^2^). The prevalence of overweight was 12.8 % at ages 16–20 and 28.4 % at ages 31–35; obesity was 7.9 % and 20.9 % at the same age groups. The main ethnic groups also showed varied patterns of obesity and overweight at the different age groups with Chinese at lowest and Orang Asli at highest risk. Level of education, employment status, physical activity and frequency of eating out were poorly predictive of overweight and obesity.

**Conclusion:**

The pattern of overweight and obesity in the 16–35 age group further highlights this as a significant period for changes in health-related behaviours. Further longitudinal research is however needed to confirm the observed pattern and investigate causal factors.

## Background

With increasing rates and links to significant morbidity and mortality from non-communicable diseases (NCDs), such as type two diabetes, cardiovascular disease and various cancers, obesity is now a urgent global health issue [1]. Indeed, obesity is a public health priority in low and middle income countries, where, in some instances, rates surpass those of wealthier nations [2] and where health systems face the complex public health challenges of both over and under-nutrition [3]. Moreover, in Asian populations, because standard Body Mass Index (BMI) thresholds for overweight/obesity (25 and 30 Kg/m2 respectively) have been linked with higher levels of body fat than other populations [4, 5] obesity-related disease burdens may be underestimated [6].

Obesity rates vary notably across South East Asia, with Malaysia and Singapore recording some of the highest levels [2]. In Malaysia, obesity rates have increased over the last 20 years [7] and this is now a critical public health issue and a priority research area [8]. In these multi-ethnic states, obesity/overweight rates vary across population groups, with the Malay and Indian ethnic groups generally recording a higher prevalence than the ethnic Chinese [7, 9–11]. Age, gender, wealth and education have also been identified as factors that influence obesity prevalence [12, 13]. The reasons for the ethnic variations however remain unclear, particularly given their similar exposure to obesogenic environments [14].

Obesity often results from the cumulative effects of years of eating patterns and physical inactivity established at a younger age. In this regard, the transition from adolescence to early adulthood is a critical period [15], with longitudinal research showing that obesity prevalence increases notably during this time [16]. During these formative years, peer influences, transition from school to higher education or employment, new found independence and exposure to new foods, behaviors and environments create a complex ecological system that adolescents navigate and that influences future behaviours.

Population-based studies on obesity and other NCD risks are beginning to build an evidence base. However, there are a number of key areas for which there remains a dearth of data. For example, to date, little research in Asia has investigated the transition from adolescence to early adulthood with regard to its relevance for the development of obesity/overweight. Drawing on data from a multi-ethnic population in rural and semi-urban Malaysia – the South East Asia Community Observatory (SEACO) – this article explores obesity and overweight amongst adolescents and young adults. The following questions are therefore addressed: what are the rates of overweight and obesity among adolescents and young adults? How do the rates vary across the different ethnic groups? How do eating habits (particularly eating outside of the home) vary across the different age groups? How does physical activity vary across the different ethnicities and age groups? What are the factors associated with BMI across these age groups? The responses to these questions will underpin any future longitudinal research on adolescents’ transition to adulthood and its influence on obesity-related behaviours.

## Methods

### Setting

SEACO is a health and demographic surveillance site (HDSS) located in Segamat District, Johor, Malaysia. Established in late 2011, SEACO covers a population of approximately 40 000 from about 11 000 households in rural, semi-urban and plantation areas. The ethnic mix of the population reflects the national proportions of Malay (60 %), Chinese (23 %) and Indian (7 %) descent, as well as gender (49 % male and 51 % female). This population is spread over five of the 11 sub-districts that comprise Segamat District

Data from the 2012 SEACO census suggest that around half of 15- to 20-year-olds migrate out of the district. In absolute terms, this is highest amongst the Malay ethnic group, however, in relative terms, the Chinese male population exhibits the highest proportion of group outmigration, with the population decreasing by more than two thirds [17]. This outmigration is linked to the transitions that they undertake between adolescence and early adulthood. Around 70 % of Malaysians attend secondary education [18], and they are required to remain an additional 18 months to gain qualifications for higher education (form six). One fifth of young people subsequently enroll in higher education [19]. Nationally, youth unemployment rates are around 10 % [20].

### Population, sample and data collection

During the initial 2012 SEACO census, all households within the five selected subdistricts in Segamat were visited to enumerate and enroll the population into the longitudinal dynamic cohort [17, 21]. A response rate of approximately 85 % was achieved across the total population. This was followed by a health baseline survey in 2013. In this article, only data on young people are reported: 16–35- year-olds, which is a range used in a number of low and middle income countries, to take account of the levels of autonomy and opportunities available within the specific development contexts [22]. The total population in this age group was 5,475.

Data were collected by a team of community-based data collectors able to communicate in relevant languages (Bahasa Malay, Chinese, English and Tamil). Data were recorded directly on Android mobile devices with survey forms designed in Open Data Kit (ODK). Data on the tablets are encrypted and are then uploaded to a secure server and encrypted again.

### Assessment tools

The health round survey comprised several modules that covered socio-demographic data, health service utilization, height and weight measurements, physical activity and self-reported health status, health service utilisation and quality of life measures. Socio-demographic data collected included: age; sex; ethnicity (Malay, Chinese, Indian, Orang Asli or Other); education (primary; secondary; tertiary) and employment.

Physical activity was measured using the WHO Global Physical Activity Questionnaire (GPAQ). The 16-item instrument, validated for the Malaysian context [23], estimates physical activity in the domains of work, transport and leisure as well as sedentary behavior [24]. The guidelines prescribed by the WHO GPAQ tool were followed to derive supplementary variables (total physical activity and the binary categories of active (>600 Metabolic Equivalent of Tasks [METs] per week) and inactive (<600 METS per week)) [25].

Participants’ height (meters) and weight (kilograms) were measured using a *TRANSTEK* scale with height gauge (GBS-721). BMI was calculated from these measures. The average number of meals eaten outside the home (per week) was self-reported. No details were collected on specific dietary intake or composition.

### Analysis

#### Body Mass Index (BMI)

For 20- to 35-year-olds, Body Mass Index (BMI) was classified using standard WHO categories: underweight <18.5 kg/m^2^; normal 18.5–24.9 kg/m^2^; overweight 25.0–29.9 kg/m^2^; obese ≥ 30.0 kg/m^2^. For 16- to 19–year-olds, the WHO gender-specific zBMI scores were used to calculate the thresholds. Calculating these cut-offs entailed taking the means of males and females values over the monthly intervals that are specified by the WHO. Therefore, for the 16- to 20-year-old age group, between the ages of 16 years 0 months and 19 years 0 months, the zBMI scores (whereby underweight < −1﻿ standard deviation (SD); normal -1SD to +1SD; overweight: +1SD to +2SD; obesity: > + 2SD) were averaged along with the standard adult BMI cut-offs between 19 years 1 month and 20 years 11 months. For this group, with both sexes combined the cut-offs were: underweight < 18.7 kg/m^2^; normal 18.7–24.7 kg/m^2^7kgm^2^; overweight 24.7–29.3 kg/m^2^; and obese ≥29.3 kg/m^2^. Data on prevalence of underweight, normal weight, overweight and obese amongst 16– to 35-year-olds are presented.

#### Physical activity

The internal consistency of the list of 16 GPAQ questions were assessed using Cronbach’s alpha [26]. All the questions had high coefficients of reliability ranging from 79 to 91 %. Hence the internal consistency of the GPAQ test scale exceeds the minimum threshold (of alpha values of 0.7 to 0.8) recommended for comparing groups [27].

#### Associations

Multinomial logistic regression models were fitted to the categories of BMI using the social and demographic factors collected as part of the health round. The m﻿odels presented are based on data from those who responded to the survey questions relevant to obesity risk, a total of 5,319 Malaysian youth.

## Results

The 16- to 35-year-old population for whom data were collected in the SEACO health round is majority Malay (72.6 % and slightly higher than the SEACO population as a whole), followed by Chinese (14.9 %), Indian (10.1 %) and Orang Asli (2.4 %). A majority received some secondary education (76.2 %) and most (64.8 %) remain unmarried. One quarter were students and just over one third were in full-time employment (34.1 %) (see Table 1).

**Table 1:** Population characteristics

	Male		Female		Male & female	
	n	%	n	%	n	%
Age (years)						
16–20	935	37.4	1021	36.2	1956	36.8
21–25	540	21.6	610	21.7	1150	21.6
26–30	518	20.7	580	20.6	1098	20.6
31–35	509	20.3	606	21.5	1115	21.0
Total	2502	47.0	2817	53.0	5319	100.0
Ethnicity						
Malay	1869	74.7	1985	70.6	3854	72.6
Chinese	373	14.9	419	14.9	792	14.9
Indian	215	8.6	323	11.5	538	10.1
Orang Asli	43	1.7	84	3.0	127	2.4
Education						
None	6	0.2	11	0.4	17	0.3
Primary	113	4.5	145	5.2	258	4.9
Secondary	1949	78.4	2077	74.2	4026	76.2
Tertiary	257	10.3	407	14.5	664	12.6
Other	160	6.4	158	5.6	318	6.0
Marital Status						
Never married	1759	73.8	1542	56.9	3301	64.8
Married	615	25.8	1113	41.0	1728	33.9
Separated	1	0.0	12	0.4	13	0.3
Divorced	8	0.3	37	1.4	45	0.9
Widow(er)	0	0.0	7	0.3	7	0.1
Cohabiting	0	0.0	1	0.0	1	0.0
Employment						
Too young	53	2.1	74	2.6	127	2.4
Student	615	24.7	719	25.7	1334	25.2
House-wife/-husband	5	0.2	668	23.9	673	12.7
Not working	239	9.6	323	11.5	562	10.6
Casual employment	27	1.1	13	0.5	40	0.8
Part-time	116	4.7	146	5.2	262	5.0
Full-time	1056	42.3	747	26.7	1803	34.1
Self employed	383	15.4	108	3.9	491	9.3
Physical activity						
Active	366	66.2	255	53.3	621	60.2
Inactive	187	33.8	223	46.7	410	39.8
Body mass index						
Underweight (BMI <18.5)	311	12.9	382	13.9	693	13.4
Normal (BMI 18.5–24.9)	1329	55.1	1422	51.9	2751	53.4
Overweight (BMI 25.0–29.9)	506	21.0	513	18.7	1019	19.8
Obese (BMI ≥30.0)	267	11.1	423	15.4	690	13.4

Sixty percent of this group were classified as active (>600 METS) and just over half classified as normal for BMI. Using standard WHO thresholds, the prevalence of overweight was significantly higher among males than in females (i.e., 21.0 % compared to 18.7 %), but obesity was significantly higher in females (15.4 % compared to 11.1 %). These differences were statistically significant (*p* < 0.001) (see Table 2).

**Table 2:** Prevalence of obesity, overweight, normal, underweight, inactive and the mean number of meals eaten outside the home according to age group and ethnicity

	Age group / years	Ethnicity				
		Malay	Chinese	Indian	Orang Asli	All
Obesity (BMI ≥30 kg/m^2a^) prevalence / %	16–20	9.5	5.3	6.0	17.6	8.4
	21–25	11.4	6.6	15.9	20.0	11.5
	26–30	17.4	11.8	15.2	21.9	16.7
	31–35	20.6	12.3	28.1	34.4	20.9
Overweight (BMI 25.0–29.9 kg/m^2b^) prevalence / %	16–20	12.0	14.4	14.3	14.7	12.8
	21–25	18.7	20.7	17.8	32.0	19.1
	26–30	26.2	13.6	26.3	53.1	25.7
	31–35	28.8	25.4	26.6	37.5	28.4
Normal (BMI 18.5–24.9 kg/m^2c^) prevalence / %	16–20	56.8	59.8	50.0	52.9	56.7
	21–25	56.6	57.9	43.9	48.0	55.3
	26–30	47.5	65.5	48.5	25.0	48.8
	31–35	45.2	60.1	41.7	28.1	46.1
Underweight (BMI <18.5 kg/m^2d^) prevalence / %	16–20	21.7	20.6	29.7	14.7	22.1
	21–25	13.4	14.9	22.4	0.0	14.1
	26–30	8.9	9.1	10.1	0.0	8.7
	31–35	5.4	2.2	3.6	0.0	4.6
Prevalence inactive (<600 METs per week) / %	16–20	47.8	47.1	44.1	32.0	46.5
	21–25	39.7	20.7	28.6	33.3	35.2
	26–30	35.9	27.6	25.0	18.8	30.8
	31–35	36.9	46.9	12.5	15.8	34.4
Mean number of meals eaten outside the home (95 % CI)	16–20	4.6 (4.3–4.9)	4.0 (3.1–4.9)	6.5 (5.9–7.2)	1.1 (0.6–1.5)	4.9 (4.6–5.2)
	21–25	5.4 (5.0–5.8)	4.2 (3.0–5.3)	9.0 (7.9–10.3)	0.8 (0.3–1.3)	5.7 (5.3-6.0)
	26–30	5.2 (4.8–5.5)	4.8 (3.4–6.1)	6.8 (5.6–8.0)	1.0 (0.0–2.0)	5.1 (4.8–5.4)
	31–35	4.5 (4.1–4.8)	2.7 (1.9–3.5)	6.3 (5.2–7.4)	0.8 (0.2–1.5)	4.4 (4.1–4.7)

Using WHO zBMI scores for 16– to 20 year-olds: ^a^ ≥ 29.3kgm^2^; ^b^24.7–29.3kgm^2^; ^c^18.7–24.7kgm^2^; ^d^ < 18.7 kg/m^2^

**Fig. 1:**
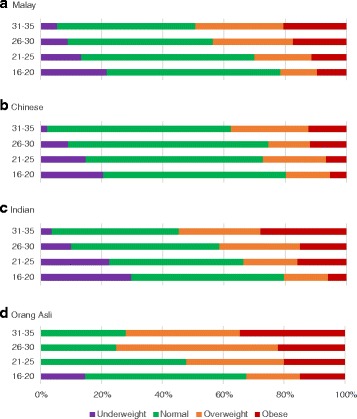
Prevalence of BMI categories amongst the different age groups by major ethnic group

**Fig. 2:**
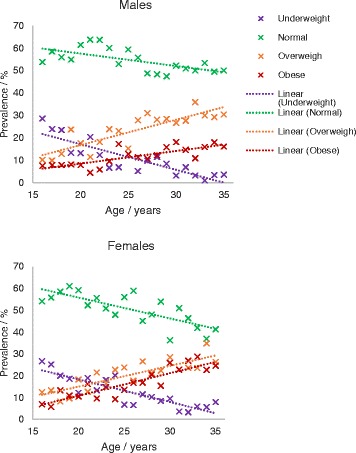
Prevalence of BMI categories amongst the different age groups by gender

BMI categories were charted across the age groups (Table 2, Figs. 1 and 2). Obesity and overweight at ages 31–35 are higher than at ages 16–20 (8.4 % compared to 20.9 %, and 12.8 % compared to 28.4 % respectively). The proportion of underweight and normal BMI is also lower in the older age groups (21.7 % at ages 16–20 versus 4.6 % at ages 31–35, 56.7 % at ages 16–20 and 46.1 % at age 31–35 respectively). Figure 2 also indicates the differences in age-specific prevalence of obesity, overweight, normal and underweight by gender.

The Orang Asli record the highest prevalence of obesity amongst the ethnic groups (22.8 %). Obesity is lowest amongst the Chinese (7.6 %). The greatest difference in obesity rates across the age groups occurs in the Indian population (6.0 % among the 16– to 20-year-olds to 28.1 % among 31– ﻿to ﻿35-year-olds). The Chinese demonstrate the lowest difference in obesity prevalence.

The relationship of obesity to physical activity and eating out are less clear. The Orang Asli reported the lowest frequencies of eating out (around once a week). The Indian youth ate out approximately six to nine times each week (Table 2). The lowest level of physical activity was recorded in the 31– to 35-year-old Orang Asli and Indians.

A multinomial logistic regression was conducted with social, demographic and behavioural factors (eating out and physical activity) using normal BMI as the base outcome for comparisons (see Table 3). The results indicate that one unit increase in age leads to an increased probability of 1.05 (*P* < 0.001) of being overweight and 1.06 (*P* < 0.001) increase of being obese. The relative risk ratios (RRRs) compare Indian, Chinese, and Orang Asli to Malay with normal BMI as the base outcome. The Orang Asli youth have double the relative risk of being overweight (*P* = 0.002) if all other variables are held constant. Being Chinese reduces the risk of overweight (by a factor of 0.74, *P* < 0.05) and the risk of obesity (by a factor of 0.46, *P* < 0.001). Indian ethnicity increases the risk of underweight (*P* = 0.002). Other factors that affect the likelihood of being overweight are marriage and employment status.

**Table 3:** Predictors of BMI among overweight and obese 16–35 year olds (with normal BMI as the reference group)

Characteristic	Unadjusted models			Base model: with all predictors			Reduced model: with selected predictors		
	RRR	[95 % CI]		RRR	[95 % CI]		RRR	[95 % CI]	
Over weight									
Age (years)	1.07***	1.0566	1.0818	1.03***	1.0111	1.0492	1.05***	1.0328	1.0650
Sex									
Male	1.00			1.00					
Female	0.99	0.8566	1.1369	0.90	0.7585	1.0661			
Ethnicity									
Malay	1.00			1.00			1.00		
Indian	1.16	0.9079	1.4724	1.11	0.8602	1.4413	1.07	0.8300	1.3821
Chinese	0.74***	0.5966	0.9068	0.86	0.6876	1.0719	0.82	0.6563	1.0141
Other	0.53*	0.2983	0.9539	0.37***	0.1884	0.7297	0.45**	0.2516	0.8158
Orang asli	2.32***	1.5143	3.5395	1.70*	1.0516	2.7548	1.86**	1.1983	2.9008
Marital status									
Married	1.00			1.00			1.00		
Not married	0.45***	0.3883	0.5214	0.67***	0.5408	0.8277	0.68***	0.5584	0.8160
Eating out	0.98***	0.9706	0.9939	0.98**	0.9703	0.9959	0.99	0.9771	1.0016
Education									
None	1.00			1.00					
Other	1.78	0.3877	8.1702	3.33	0.6942	15.9796			
Primary	3.66	0.8043	16.6475	3.19	0.6815	14.9765			
Secondary	2.43	0.5475	10.8000	2.72	0.5919	12.5298			
Tertiary	2.61	0.5810	11.7014	3.32	0.7129	15.5032			
Employment									
Full-time	1.00			1.00					
Student	0.45***	0.3630	0.5491	0.66***	0.5004	0.8783			
House-wife/-husband	1.18	0.9483	1.4623	0.93	0.7132	1.2116			
Not working	0.54***	0.4051	0.7112	0.69*	0.5053	0.9412			
Casual jobs	1.32	0.6372	2.7140	1.15	0.5360	2.4560			
Part-time	1.02	0.7276	1.4344	1.14	0.8073	1.6180			
Pensioner	0.00	0.0000	.	0.00	0.0000	.			
Self employed	1.24	0.9779	1.5744	1.24	0.9609	1.5998			
Too young	0.41***	0.2195	0.7691	0.66	0.3321	1.2981			
Total physical activity	1.00	0.9992	1.0013	1.00	0.9988	1.0012			
Obese									
Age (years)	1.08***	1.0640	1.0939	1.06***	1.0348	1.0794	1.06***	1.0406	1.0785
Sex									
Male	1.00			1.00					
Female	1.52***	1.2847	1.8020	1.23*	1.0023	1.5055			
Ethnicity									
Malay	1.00			1.00			1.00		
Indian	1.26	0.9627	1.6460	1.18	0.8917	1.5691	1.21	0.9123	1.5930
Chinese	0.46***	0.3485	0.6188	0.55***	0.4054	0.7349	0.56***	0.4173	0.7499
Other	0.16***	0.0514	0.5230	0.11***	0.0350	0.3757	0.14***	0.0442	0.4547
Orang asli	2.29***	1.4289	3.6774	1.34	0.7833	2.2757	1.81*	1.1112	2.9555
Marital status									
Married	1.00			1.00			1.00		
Not married	0.43***	0.3661	0.5155	0.85	0.6587	1.0936	0.73***	0.5830	0.9045
Eating out	0.96***	0.9451	0.9746	0.98***	0.9595	0.9912	0.97***	0.9538	0.9846
Education									
None	1.00			1.00					
Other	0.46	0.1542	1.3978	1.07	0.3285	3.5024			
Primary	1.21	0.4146	3.5511	1.32	0.4249	4.1195			
Secondary	0.66	0.2355	1.8690	0.89	0.2930	2.6764			
Tertiary	0.53	0.1828	1.5295	0.84	0.2682	2.6047			
Employment									
Full-time	1.00			1.00					
Student	0.47***	0.3641	0.6126	0.83	0.5893	1.1827			
House-wife/-husband	2.08***	1.6485	2.6299	1.43*	1.0666	1.9125			
Not working	1.11	0.8342	1.4676	1.33	0.9659	1.8338			
Casual jobs	0.79	0.2687	2.3427	0.78	0.2592	2.3545			
Part-time	1.08	0.7102	1.6296	1.13	0.7373	1.7291			
Pensioner	0.00	0.0000	.	0.00	0.0000	.			
Self employed	1.15	0.8492	1.5505	1.12	0.8113	1.5347			
Too young	0.81	0.4366	1.4870	1.38	0.7004	2.7194			
Total physical activity	1.00	0.9990	1.0015	1.00	0.9996	1.0023			

*RRR* relative risk ratio, *CI* confidence interval; * p<0.05; ** p<0.01; ***p<0.001

Age, ethnicity, marital status and employment status are therefore statistically significant predictors of BMI among the population of Segamat 16– to 35–year-olds. Being physically inactive did not however produce a significant RRR value for any of the BMI categories relative to normal; nor did level of education.

## Discussion

The levels of obesity and overweight across the 16– to 35-year-old age group of SEACO participants further highlight the significance of this life stage in terms of trends in BMI. Relatively little obesity-related research in Malaysia has focused on young people and few data are directly comparable with those presented above. Furthermore, because of differences in study design comparisons with the available studies of young Malaysian’s obesity rates, diets and activity habits (e.g., [28, 29]) are of little value. Nonetheless, the SEACO data are in line with the increases in obesity prevalence reported in a variety of studies across Malaysia since the mid-1990s [7]. The pattern amongst the Orang Asli is particularly pronounced but this may be a result of the small number of respondents: a total of 123 respondents provided information on height and weight for the health round.

The increasing obesity prevalence in Malaysia has been explained in terms of the concurrent rise in national wealth, urbanization and industrialization [30]. Although often termed a “disease of affluence”, cross-national comparisons indicate that the association between national wealth and obesity prevalence is more nuanced [3]. This emphasizes the need to investigate Malaysia’s obesity epidemic in its own terms, exploring both rural and urban environments to identify the obesogenic factors [31]. Several of these obesogenic factors are in evidence in Segamat as in many other areas of Malaysia. For example, Western fast food outlets are a growing enterprise [30], with, amongst others, McDonalds, KFC and Pizza Hut popular eateries whose advertising is often aimed at young people. Furthermore, if considered expensive, cheaper local imitations (for example, Ramly burgers) are widely available. The ubiquity of fried food – whether, local, Western or a mix – is also reflected in patterns of cooking oil consumption: global trends, whereby increased vegetable oil consumption contributed to rises in calorie consumption between the mid-1980s and 2000s [32], are particularly apparent in Malaysia, where per capita consumption of fats and oils – particularly palm oil – is among the highest in Asia, [33]. Indeed, the economic importance of palm oil to Malaysia is keenly visible in Segamat, where palm plantations dominate the landscape.

Levels of physical activity are comparable with data from other studies using the GPAQ in Malaysia [34]. Again, research in this area is limited, with the last population-based survey from 2002 to 2003 indicating low levels (14 % of respondents) of physical activity [35]. This inactivity is partly attributed to the primacy of motor vehicles and motorcycles for transport. Indeed, other data from the SEACO surveys emphasize the ubiquity of car ownership, with at least one vehicle in every household. Observations in Segamat also lay bare the lack of pedestrian and cyclist-friendly infrastructure. Few journeys are therefore taken on foot or by bicycle.

In terms of eating out, a recent review identified associations between eating out and higher total energy and fat intake [36]. The SEACO data however suggest little connection between overweight or obesity, and eating out. In this context, (as it may be in others), the relationship between eating out and overweight/obesity is therefore probably more complex. This resonates with studies that have drawn attention to the significance of the type of restaurant/fast food outlet, rather than just *eating out* [37]. Research elsewhere in South East Asia has also highlighted that eating out does not necessarily entail higher intake of fat and energy [38].

### Strengths, limitations and further research

Broader inferences of prevalence from this study are limited by the focus on a single predominantly rural community (albeit with some semi-rural areas); SEACO was set up to explore the nature of relationships and seek detailed explanations for changes to population health and wellbeing, and not necessarily to produce nationally representative epidemiological data. Nonetheless, cross-sectional data generated from the platform provide a detailed picture of the whole community as opposed to samples of populations. In addition, relatively little obesity-related research has been undertaken in Malaysia, or indeed in the region, that focuses on adolescence and early adulthood. Small studies have been undertaken in targeted small student groups within university campuses with significantly less generalizability [28, 29].

The data presented on food and activity habits are self-reported and therefore subject to potential bias. Although the data on physical activity compare well with another study conducted in Malaysia [34], further research is needed, ideally using validated techniques and potentially innovative approaches, for example, taking advantage of the commonness of mobile phones to log food and physical activity habits.

The data presented are limited by their cross-sectional nature and the possible impact of cohort effects. Although it is likely that similar trends would be observed in SEACO’s cohorts, further research is needed to demonstrate this and to investigate the full impact of the transition from late adolescence to early adulthood on overweight and obesity.

## Conclusion

The increased overweight and obesity at older ages in the 16- to 35-year-old group illustrates that this is a significant period for changes in health-related behaviours. The changes in obesity and overweight are particularly stark because this is a predominantly rural context and in such areas it is often assumed that there are more opportunities for healthier food options and physical activity than in urban areas. Further longitudinal (qualitative and quantitative) research is however needed to confirm the observed pattern and investigate thoroughly the causal factors.

## Abbreviations

BMI: Body mass index
CI: Confidence interval
GPAQ: Global physical activity questionnaire
NCD: Non-communicable disease
ODK: Open data kit
RRR: Relative risk ratio
SD: Standard deviation
SEACO: South East Asia Community Observatory
NCD: Non-communicable disease
